# Flights and Perchings of the BrainMind: A Temporospatial Approach to Psychotherapy

**DOI:** 10.3389/fpsyg.2022.828035

**Published:** 2022-03-24

**Authors:** Aldrich Chan, Georg Northoff, Ryan Karasik, Jason Ouyang, Kathryn Williams

**Affiliations:** ^1^Graduate School of Education and Psychology, Pepperdine University, Malibu, CA, United States; ^2^Center for Neuropsychology and Consciousness, Miami, FL, United States; ^3^Faculty of Medicine, Centre for Neural Dynamics, The Royal’s Institute of Mental Health Research, Brain and Mind Research Institute, University of Ottawa, Ottawa, ON, Canada; ^4^Mental Health Centre, Zhejiang University School of Medicine, Hangzhou, China; ^5^Centre for Cognition and Brain Disorders, Hangzhou Normal University, Hangzhou, China

**Keywords:** spatiotemporal, psychotherapy, neuroscience, consciousness, interpersonal neurobiology, treatment, brain, mind

## Abstract

This article introduces a process-oriented approach for improving present moment conceptualization in psychotherapy that is in alignment with neuroscience: the *Temporospatial movements of mind (TSMM)* model. We elaborate on seven temporal movements that describe the moment-to-moment morphogenesis of emotional feelings and thoughts from inception to maturity. *Temporal* refers to the passage of time through which feelings and thoughts develop, and electromagnetic activity, that among other responsibilities, bind information across time. Spatial dynamics extend from an undifferentiated to three dimensional experiences of emotional and cognitive processes. Neurophysiologically, *spatial* refers to structures within the brain and their varying interactions with one another. This article culminates in the development of an atheoretical temporospatial grid that may help clinicians conceptualize where patients are in their cognitive and emotional development to further guide technique.

## Introduction

Activity in the brainmind moves through the passage of time and space with varying speed and direction. Phenomenologically, this activity may decelerate as it focuses and accelerate in transition from one experience to the next. In 1890, William James compared the rhythm of a bird’s “flights and perchings” (p. 243) to exemplify the transitive and formative aspects of the *stream of consciousness*. An important part of psychotherapy is the formulation of a ‘flight path’ whereby the therapist can facilitate the maturation of psychological experience. Yet this process of maturation has multiple ‘destinations’ and may occur on a much smaller time-scale than generic treatment plans can offer. Small inputs may result in large changes within a single session. The type and timing of an intervention may be just as important as their delivery and relevance.

The field of psychotherapy is vast, with several effective theoretical orientations emerging in reaction to one another; similar to the parable of blind men agreeing and disagreeing about their experiences as they examine specific parts of an elephant. Studies suggest that regardless of the approach, psychotherapy may facilitate neuroplasticity impacting brain structure and function (Cozolino, 2017; Kalsi et al., 2017), and furthermore, the positive outcome of treatment is quite comparable amongst the varying approaches (Cuijpers et al., 2019). The most profound factor facilitating positive change was not identified as any particular theory but rather the quality of the therapeutic relationship. One commonality that all approaches share is the necessity to consider the client’s development of feelings and thoughts. Development occurs as a process which can be conceptualized through time and space, or in the terms of James, Flights and Perchings.

In order to address individual differences within the vast spectrum of human experience, clinical practice has opened up to incorporate eclectic paradigms. The integration of various conceptual approaches often results in a particular form of treatment whose fundamental assumptions knit together consonance amongst theories, or a singular conceptual approach that may integrate techniques from other theories. With the abundance of theoretical orientations and techniques, therapists are often inundated with their applications leading to an increased risk of implementing them in the wrong context. One way to decrease this risk is to provide a process-oriented framework that may inform what techniques may be useful in any context.

The purpose of this paper is to build, explicate and refine Chan’s (2021) conceptual model that addresses Allan Schore’s (2019) suggestion to direct “therapeutic technique toward the elevation of emotions from primitive presymbolic sensorimotor level of experience to mature symbolic representational level, and a creation of self-reflective position that can appraise the significance and meanings of these affects” (p. 280).

## Influences

Chan’s framework sought to update Wilfred Bion’s grid, in accordance with modern day neuroscience and theories of self, especially from the work of Antonio Damasio, Jaak Panksepp, and Carl G. Jung. Each phase was termed a “movement” to emphasize process as espoused by several influential thinkers, from philosophers such as Heraclitus, Charles Sanders Peirce, and Alfred North Whitehead to Psychologist/Philosophers including William James and John Dewey, to Psychoanalysts Wilfred Bion, and Donald Winicott.

Moment-to-moment transitions allude to the dimension of time. This iteration adds a dimension of Space which develops from the work of James Grotstein (2002), as well as a more formal recognition of emotional evolution, especially from the work of Jaak Panksepp. This conceptualization will be formally known as the *Temporospatial Movements of Mind (TSMM)* model; with the mind being defined as an embodied and relational process that regulates the flow of energy and information (Siegel, 2012). Characterizing human subjective experience and objective processes as being in perpetual relational flux informs us that even the final movement undergoes reconsolidation, returning to the first movement. To improve relatability and application, concrete examples will be provided throughout each section. Finally, a visual representation and an updated “grid” with space on the *X*-axis and time on the *Y*-axis will be provided for therapeutic use.

## Temporospatial Dynamics in Neuroscience

The ideas of time and space are often attributed to the fields of physics and perception. What is less known, is that our brains also operate amongst the background and foreground, anchored in unique signatures of their own inner time and space. There is a temporospatial exchange between the brain and the world. Time and space may be considered the “shared currencies” between objective reality and our subjective experience (Northoff et al., 2020b). The neuroecological and temporospatial model is a paradigm formulated by Northoff (2018) that seeks to understand how our brain-mind’s align with the phenomenal world. This is described as “temporo-spatial alignment”, which is one of the four key temporo-spatial mechanisms of the brain’s neural activity for consciousness (i.e., Temporo-spatial theory of consciousness (TTC), Northoff and Huang, 2017; Northoff and Lamme, 2020).

A key feature of the temporo-spatial approach to brain-mind by Northoff is that the brain’s inner time and space exhibit an elaborated structure that is topographical and dynamic (Northoff et al., 2020a). Structural and functional connectivity within and between areas of the brain generate a spatial topography visible by neuroimaging such as magnetic resonance imaging (MRI) and functional magnetic resonance imaging (fMRI), While electromagnetic fields emerging from the brain’s dynamic activity form a temporal structure which can be measured through electroencephalograms (EEG), and/or magnetoencephalograms (MEG).

Brown (2021) estimates that we have 100 mind/brain states within 10 s, each which overlaps and builds upon one another, similar to waves combining to form a wavefront. Damage to an area of the brain, such as the left hemisphere, may not destroy a particular function, but rather disrupt the process of the function. An aphasic subject who mistakes a table for another furniture piece, experiences the disruption of brainmind processes that serve to narrow down a category into a specific item. Put simply, it is not the destruction of a 1–1 correspondence, there is no specific brain area for “table,” but the process that enables this identification. This demonstrates how a process-oriented framework provides an interpretation more aligned with the empirically validated understanding of the brainmind.

From the spatial perspective, there have been a surplus of studies identifying robust changes in the structure and function of the brain in victims of posttraumatic stress disorder (PTSD) (see Chan, 2016 for extensive review). The neural networks’ overall dysfunctions can be characterized as such: default mode network activity (e.g., correlates to introspection) is interrupted by the salience network and central executive network activity (e.g., correlates to goal directed activity) exhibits intrusions from the default network. This is in line with several studies identifying hyperactive amygdala activation during resting state which contributes to hyperarousal as well as decrements in specific cognitive functions when an individual is focused on goal-directed activity (Chan, 2016).

The group around Northoff (Duncan et al., 2015) provided three levels of analysis that supports the idea that the brain’s temporospatial structure is highly defined by early experiences. At the first level, they concluded that there are higher levels of complexity (i.e., entropy) in signals across time and space in individuals who suffered from early childhood trauma. One notable area related to self-processes is the perigenual anterior cingulate cortex (PACC), which exhibited inconsistent signaling that continued into adulthood. There was a significant correlation between childhood trauma and levels of entropy. In this way the brain’s intrinsic activity implicitly encoded temporospatial features, generating “temporospatial memory” (Northoff, 2016, p. 229) expressing the impact of traumatic interpersonal experiences. An abundance of research on the influence of attachment on adult relationships may also be supportive of this framework (Siegel, 2019). Areas such as the orbital medial prefrontal cortex, and its connections to the limbic system have been identified as core regions for attachment based responses (Schore, 2019).

Mucci’s model of interpersonal trauma (Mucci, 2018; Mucci and Scalabrini, 2021) suggests that early relational trauma (ERT) that occurs as a consequence of poor attunement between the mother and child can lead to psychopathology that renders the mother unable to provide the care required for the child’s optimal development. Lack of attunement may lead to affect dysregulation which can leave the child vulnerable to drug and alcohol abuse, eating disorders, interpersonal difficulties, and impulsivity. ERT has been found to be associated with decreased functional connectivity within the DMN in the resting state, suggesting an unstable and insecure sense of self. Furthermore, children who suffer severe abuse may develop a vulnerability toward developing disorganized attachment. In tandem, dissociative symptoms may lead to conflictual internal experiences, such as simultaneous identification with the victim and perpetrator. Experiences of victimization include: low self-esteem, blame, shame, and guilt, while an internal persecutor (unconsciously) experiences violence, hate, and aggressiveness (often toward one’s own body). An identification with the aggressor is thought to reflect a dissociative defense mechanism which allows the individual to avoid experiencing the overwhelming affects associated with the trauma (Mucci, 2018). This victim/persecutor dissociation may be reflected in severe personality disorders, such as borderline personality disorder or narcissistic personality disorder.

Mucci also refers to “massive trauma”, caused by massive calamities such as genocides and war. Individuals subjected to these massive traumatic events may suffer psychopathological effects on a personal level and inadvertently contribute to the first and second level of traumatization in their offspring by not being able to provide the attunement and attachment necessary for their offspring’s optimal development. Massive trauma may lead to an intergenerational transmission of trauma whose effects percolate down to the second and third generations of trauma survivors. The transmission of such interpersonal trauma finds expression in alterations of the neuroecological layer characterized by changes in the brain’s spatiotemporal structure and features.

On the second level, Duncan et al. (2015) encountered alterations in glutamate, a neurotransmitter playing a central role in neurodevelopment, more specifically lower levels in the PACC. Thirdly, they found that when anticipating an aversive stimulus there was lower activity in the right anterior insula and motor cortex in individuals who suffered from early childhood trauma. This anticipatory response correlated significantly with degree of entropy and glutamate as well. Another study conducted by Nakao et al. (2013) also identified alterations in infra-slow frequency fluctuations (IFF) in neural activity (0.01–0.1 Hz) in the medial prefrontal cortex, another area highly correlated to self-processes. In particular, they found that higher levels of childhood trauma correlated to lower power in IFF in the aforementioned region. Northoff (2016) concludes “the balance between the environmental life events” “external disturbing power” and the brain’s “internal modifying power” may determine the degree to which “life events as perceived” deviate from the “life events as real” and consequently the degree to which life events are encoded and subsequently perceived as more less traumatic” (P. 236).

## Temporospatial Neuroscience of Brain and Self

Temporospatial dynamics may be the common intrinsic feature which connects brain and psyche. Instead of characterizing the brain and self by specific functions of single regions/networks, temporospatial neuroscience explores the spatial topography and temporal dynamics of neural activity and subjective mental contents. Spontaneous activity is topographically identified within interacting networks like default-mode, salience, and central executive network and is temporally characterized by the balance between infraslow, slow, and fast frequencies (Northoff and Scalabrini, 2021).

Specifically, longer and more powerful slower frequencies nest the faster frequencies which allow for correlation of varying timescales to provide temporal stability (i.e., long range temporal correlation). A kind of temporal or dynamic memory forms in which past neural patterns influence future dynamics outside of our conscious awareness. Abnormal temporal integration of stimuli may contribute to phenomenological experience of discontinuous sense of self observed in disorders like schizophrenia (Northoff et al., 2020b).

The neural correlates of the self can be conceptualized under a spatially nested hierarchy in which brain regions (i.e., bilateral insula), of lower layers (e.g., interoceptive processing), are recruited again and complement additional regions within higher layers of the cognitive self (Qin et al., 2020; Northoff and Scalabrini, 2021). Conducting a large-scale meta-analysis, Qin et al. (2020) observed three levels of self. The interoceptive self is the most basic layer and recruits interoceptive regions like the insula and thalamus; these regions are also recruited by the exteroceptive–proprioceptive self which also involves the bilateral temporo-parietal junction, the premotor cortex and the medial frontal cortex. Finally, these regions are also recruited during the mental or cognitive self plus the typical midline regions of the default-mode network. Together, this amounts to a three-layer nested hierarchical structure of the intero-exteroceptive and mental self.

Findings suggest the insula in particular is highly involved in self processing and is at the center of interoceptive processing, which could be foundationally relevant for higher-order self-processing (Qin et al., 2020). Different subdivisions of the insula were found to be particularly relevant in different functioning in the mediation of interoceptive signals. From the posterior to anterior perspective, the insula presents sensory signals ranging from objective to subjective levels of processing. In this progression, subjective feeling states are conveyed by the anterior insula which may represent a neural core explaining an individual’s awareness of bodily self. The insula’s activation in all three levels of self-processing proposes that it is still necessary for integration between external environmental signals with recognition of self.

The insula may also play a key role in narcissistic personality disorder, in which individuals tend to lack empathy in response in social relationships and environments. Although direct neural underpinnings of this are yet to be clear, there is evidence that core regions of the brain associated include the anterior insula, as well as the left inferior frontal cortex, premotor cortex, and the dorsolateral prefrontal cortex. The anterior insula is considered more related due to its relation to one’s focus on the self as mentioned above. Neuronally, studies show that individuals with high narcissistic traits psychologically produce increased scores of alexithymia, general psychopathology, and depression (Fan et al., 2010).

Research from Scalabrini et al. (2021) implicates the right insula as having more topographic and dynamic features when compared to the left. The right exhibits a higher degree of integration with other regions from an interoceptive and exteroceptive perspective and notably with the recognition to the three layers of self-processing. The right insula proves to be an ideal node for the layers of the confirmation of self-integration at a higher order state for temporal continuity.

The right insula’s high degree of functionality guarantees interoceptive, exteroceptive, proprioceptive, and cognitive or mental-self integration. SCDF hypothesize that key functioning of the right insula involves spatial nestedness on a neuronal level which also precipitates psychologically. This may constitute a shared feature between neuronal and mental understandings of the self and self-processing.

**FIGURE 1:**
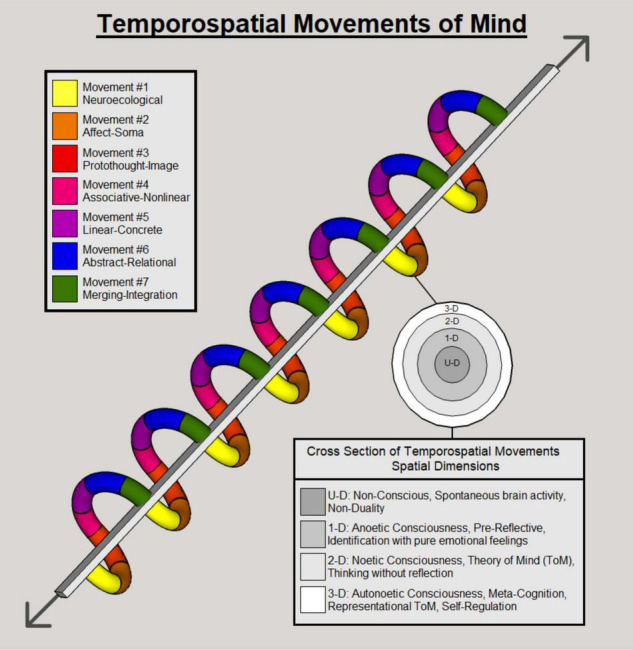
This is a visual representation of the temporospatial model to refer throughout the paper. It is followed by sections describing each spatial dimension and temporal movement in depth. For further clarification, each ring composes a full temporospatial cycle. The end (Merger-Integration) is reconsolidated into the beginning (Neuroecological), as the beginning builds upon the end (where green and yellow meet). They are iterative in nature, and can move in either direction (i.e., regression, progression). Temporal cycles are coded to corresponding colors, a cross section of which reveals their volumetric composition as spatial dimensions. Spatial dimensions are illustrated as rings to a tree trunk, colored in light gray. Note that spatial volume and temporal movements form a single stream, and each phase within respective cycles are nested within one another. Artwork by Daniel Icaza.

These three layers of self are extremely important for psychotherapy (Northoff and Scalabrini, 2021). Different mental health changes may correlate with different balances or imbalances between the three layers of self. For instance, social anxiety disorder reflects decreased midline DMN activity and thus an unstable mental self, which manifests as anxiety. That, in turn, renders the lower intero- and exteroceptive layers in these subjects as relatively stronger. This relative imbalance manifests as an increase in interoceptive awareness of the body. In this context, psychotherapeutic efforts need to address these imbalances between the three layers of self and reset them by re-establishing their hierarchical nestedness (i.e., the relative balances among each other). Figure 1 (below) is an artistic rendering of the temporospatial model, which may be of assistance in its conceptualization.

## Spatial Dynamics in Psychology

How do we characterize space and time in the mind and in such a way that it is beneficial for therapeutic use? Psychological theories have often used the metaphor of space in language. For example: object relations theory conceptualizes the mind as housing representations of people and their relationships, and self-psychology speaks about psychological structures that require rehabilitation. Addressing these questions leads us back to the brain’s spontaneous activity.

The brain’s spontaneous activity continuously attempts to synchronize with its external environment, corresponding to how someone relates to the world. Its inner time and space can modify how information from the external world is encoded and this occurs through what Northoff (2016) calls “active modification by amplification or attenuation” (p. 233) This demonstrates continual reciprocity between brain and environment. The world and our brains are not static but in continual dialog with one another, better characterized as dynamic, continuous and process-oriented. This is expressed in the key relevance of temporo-spatial alignment of the brain’s spontaneous activity to its external environment (Northoff and Huang, 2017; Northoff, 2018). Such temporo-spatial alignment operates, as in its words, through time and space shared by the brain and environment – how does the temporal dynamic of, for instance music, match with the temporal dynamic of the brain’s ongoing spontaneous activity?

The logician, mathematician and philosopher Peirce et al. (1892/1992) thought space and time are continuous and that mental events are no exception. In his own words, feelings are subject to “inhesion,” (p. 324) and if “space is continuous, it follows that there must be an immediate community of feeling between parts of mind infinitesimally near together” (P. 325). Moreover, the language humans typically use in everyday experience is defined by space, for example: whenever there is tension or an argument amongst individuals, a common reply may be “I need some space.” This may literally mean needing some physical distance but more often than not it implies psychological space in order to process issues.

When we consider dreams, one must wonder where do these dreams occur? When we engage in imagination, although images are not perceived they are experienced within the stream of consciousness. Where is that happening precisely? Meares (1984) considers it “virtual space,” as “inner vision cannot be located in a material way” (P. 163). He further argues that there is a constriction of space in states of anxiety and for those who suffer from narcissistic personality disorder. Anxiety is often characterized by a “limbic hijack” when emotions overpower frontal functioning and there becomes less space for one to respond within, one may become reactive as opposed to responsive.

In narcissism, one is restricted toward a “‘selfish center,’ and the world of material objects” (P. 166). In psychotherapeutic practice, Meares (1984) asserts that every time an association is formed and a new narrative constructed, there is an expansion of psychological space. There is now more room for one to think about other things. Notably, he concludes that the nature of psychological space is highly interpersonal and related to increasing levels of empathy. This parallels neuroecological findings described above, with increasing levels of entropy in trauma victims and their associated symptoms.

In our particular view, we use space as conceptualized in physics as an analogy. In particular, physics considers that we exist in a multidimensional universe. There is an undifferentiated dimension, characterized by implicit experience and anoetic consciousness followed by three dimensions that allow for increased complexity metaphorically characterized as length, width and height. In this conceptualization we consider four dimensions of space. Grotstein (2002) introduced the conceptualization of dimensions related to psychic space, however he includes a “null dimension,” to signify “infinite space and timelessness” (p. 85). In this model, label this the U-dimension representing an undifferentiated state between the internal and external world.

The spatial component of the TSMM operates from the framework proposed by Vandekerckhove and Panksepp (2009), which explicates anoetic, noetic and autonoetic phases of consciousness. The following (Table 1) is a brief summary of what these levels entail.

**TABLE 1:** Three phases of consciousness.

Anoetic consciousness	Involves “implicit procedural, sensory and affective memory and on the conceptualization and empirical foundation of raw affective consciousness of Panksepp, and primal perceptual consciousness…” (Vandekerckhove and Panksepp, 2009, p. 1018). It is defined by its unreflective nature. Individuals may experience the “body and world with sensory perceptual immediacy and affective intensity” (P. 1021).
Noetic consciousness	Beginning around age 2, infants develop a sense of self which is determined through the mirror experiment. In this moment, the child is now aware and “at a *knowing* level that the recognized image is of him or herself, with a sense of self belongingness…” (p. 1024). Associated with this level of consciousness is the emergence of the semantic memory system. The semantic system is atemporal and aspatial, organizing factual knowledge about the world in the ‘present.’ Factual knowledge allows one to operate and function in the world but still lacks a dynamic personal relationship with the world.
Autonoetic consciousness	Involves explicit memory systems and episodic (i.e., autobiographical memory). They are now capable of mental time travel into the future to simulate potential events and into the past to retrieve memories that are personally significant. This is where the notion of recursion begins. Narratives are formed and humans are capable of reflecting and learning from their experiences and experiences of others.

With this in mind, the therapist ideally helps facilitate higher levels of incorporation from preceding dimensions into proceeding ones. These developmental shifts in psychological territory mimic the neurodevelopment of humans from infancy to adulthood and rest on a spectrum. Moreover, overlapping regions may enable a transitory shift from one dimension to a novel array of psychological experience, and there will always exist the potential for regressive movement back to earlier phases. A general term expressing the enlargement of conscious experience, which includes higher levels of dimensional thought, will be termed *spatial dilation*. Another term of interest is that of *resistance*, which may range depending on the spatial dimension achieved. We introduce three degrees of resistance which will be explicated throughout. Finally, each one of the spatial dimensions may be characterized by the degree to which an individual’s conscious self is actively or passively involved in their behavioral expressions due to unconscious processes and actively involved in self-regulation. While certain interventions or approaches will be mentioned in transitive portions, it is important to note that these are by no means exhaustive, and only an attempt to illustrate examples.

## Undifferentiated Dimension (U-Dimension)

This dimension is characterized by interactions between undifferentiated psychosocial experiences from early life and genetic imperatives; an operation comparable to how humans were prior to having an explicit sense of self, around the age of 2 (Rochat, 2003). It corresponds to implicit systems, neuroanatomically correlated to right hemispheric lateralized structures and unconscious within the autonomic nervous system and body (Schore, 2019). Moreover this is in congruence with the work of Panksepp and Biven (2012), who would refer to unconscious secondary emotions to the upper limbic system.

One example would include individuals who suffer from schizophrenia. Such individuals may experience disruptions in neural synchrony with the environment, resulting in misperceptions in causal relationships (Northoff, 2016). They may also experience positive hallucinations and/or delusions, a major correlate of which is an overactive dopamine system in the mesolimbic pathway and more recently, the dorsal striatum in the basal ganglia (McCutcheon et al., 2019). Here we have a classic example of internal stimuli manifesting in the external world. Individuals with delusions may have a firm belief about others which are anchored in a false conviction. They may become aware of hallucinations and delusions in higher levels of the temporospatial movement. This dimension may be compared to Kernberg’s most severely disorganized level of personality organization, the psychotic level (Lenzenweger et al., 2001). Such patients exhibit impaired reality testing in which they are unable to differentiate self from others and intrapsychic stimuli from the external environment.

Attachment based trauma has also been associated with loss of coherence in spontaneous brain activity (Nakao et al., 2013). It is well known in psychodynamic work that early childhood trauma, especially from primary caregivers, may result in dysfunctional patterns of relating. These patterns are largely unconscious, and come as no surprise, given that 65% of the brain volume develops after birth, with a large proportion of the adult brain being met by 6–7 years of age (Gilmore et al., 2018). As fundamental relational circuits are established in implicit systems, so are they re-enacted throughout the individual’s adult life. Thus, in this dimension there is no resistance to affect, and they are simply expressed as implicit operations. The individual is completely passive and unaware of psychological challenges, and maladaptive behavioral expressions. Behaviorally, we find phenomena such as repetition compulsion, projections, projective identification, transference, and acting out.

## Transitive UD-1D

This transition comprises the groundwork of all psychotherapy. The establishment of a trusting relationship sets the conditions necessary for healing to occur. Alongside this process is the conversion of feelings into language. Rogerian paraphrasing may assist with the client’s feelings of being understood, and help with the organization and clarity of challenges. As trust is established, and affective laden mental contents enter from non-conscious processes into consciousness with higher levels of organization, there is a natural dilation of inner space. The clinician clarifies and confronts defenses, drawing awareness to their existence. For severe conditions, medications, TMS, ECT may all be necessary for the stabilization of neurochemical imbalances and electromagnetic coherence.

### First Dimension

One-dimensional thinking and emotional experience are by and large anoetic or preconscious, which has been associated with the orbitofrontal-limbic network (Schore, 2019). This implies decisions based on pure feeling and entails a higher potential for contents to rise into consciousness. There is a subtle distinction between subject and object, thus lacking in differentiation. The client is capable of pre-reflective thinking. We might assign *length* as its descriptor. The client vacillates between unconscious processes and awareness of their psychological existence. For example, individuals who suffer from borderline personality disorder often experience the extremes of love or hate, good or bad, with no shades of gray. They can move in the direction of love and immerse the clinician with flattery or enter the session with hate, berating the clinician. One might consider this the paranoid-schizoid position in a Kleinian approach. The individual’s thoughts are still fused or identified with primary emotional processes and non-conscious secondary emotional processing.

It is in the first dimension we find the inception of awareness in relation to defense mechanisms. To be more specific, when one identifies with a particular emotion and is unable to think through it, these emotions are externalized, often onto another person. In this scenario, the therapist is meant to help the patient first become aware of these externalizations and help them represent the content in such a way that they can take ownership and responsibility. For example: if we consider an individual who suffered from sexual or physical abuse by a parent, they may transfer similar thoughts and feelings to a same-sex authority figure, such as the clinician. These inner events may be operating implicitly, likely characterized by entropic spontaneous activity in the brain. Feelings of distrust and anger may emerge and be directed at the clinician without further thought. An individual’s thoughts and decisions are thus recruited to fulfill demands made by primary emotional processes (Panksepp, 1998; see Table 3). Thus, resistance of the first degree signifies a low threshold, yet there is a gradual reduction of emotional strength, and regulation as they are labeled (Lieberman et al., 2007), organized into an increasingly coherent narrative and made aware. The individual is passive-active, meaning psychological experiences and behavioral expressions are still being recruited by primary emotional systems, though secondarily active, with increasing awareness.

## Transitive 1D-2D

With increased awareness of unconscious processes, and greater insight into their psychological disposition and behaviors, there is a greater capacity for self-representation and social cognition. Successfully facilitating the incorporation of another individual’s perspective (such as in mentalizing) potentially reduces the strength of affective valence and results in spatial dilation. Other approaches such as dialectical behavioral therapy may also be of assistance as emotion regulation and distress tolerance are enhanced.

### Second Dimension

Two-dimensional thinking and emotional experience afford movement in two directions, length and width. There is clearer differentiation between themselves (length) and others (*width*). The added component of others enables self-representation (Uddin et al., 2005). This corresponds to a noetic dimension, whereby there is a knowing of habitual patterns, but not yet the capacity to subvert their influence. There is an awareness of primary process emotions in action but a lack of awareness of secondary process emotions. There may be a theory of mind but not yet representational theory of mind (Saxe, 2006). For example, in the clinical context, a borderline patient gains the capacity to consider that another person may have another perspective, but this perspective is incapable of being represented resulting in biased responses. Similarly, clients under Otto Kernberg’s borderline personality organization may have intact reality testing but still engage in immature defense mechanisms owing to a fragmented sense of self (Kernberg, 1968). Resistance is of the second degree, or moderate, in this dimension, as the client is able to tolerate the psychological existence of maladaptive paradigms, but still unable to incorporate insight into their behaviors. In light of this challenge, the individual is considered active-passive, signifying that awareness has increased to the extent that the individual is actively working through maladaptive psychological representations, though still experiences relapses with incongruent behaviors. Neurologically, this may be seen as a failure of the orbitofrontal cortex to effectively inhibit an internal behavioral response, and the necessity to continue developing descending inhibitory fibers (Rudebeck and Rich, 2018).

## Transitive 2D-3D

Spatial dilation expands as transferential dynamics are successfully interpreted, wider perspectives achieved, and the empathic relationship internalized. Other approaches that might be of assistance may be the use of socratic questioning (CBT) to cover “blind spots,” awareness of triggers alongside behavioral plans to assist with consolidating beneficial habits (CBT), increased non-verbal awareness (Gestalt), and self-soothing through mindfulness-based practices.

### Third Dimension

To operate from three-dimensional thinking and emotional experience, we include length, width and *height*, which represents metacognition and autonoetic consciousness (i.e., consciousness enabling mental time travel). An individual becomes conscious of conflicting motives and emotions and they can work on the ground of ambivalence. One might compare this to the depressive position in Kleinian approaches, or the achievement of object constancy in object relations, accepting that the people may have both positive and negative qualities. This form of thinking may facilitate a process of healing. Such an achievement allows one to function free of the impact dealt to them from their negative experiences, as they can represent them in an alternate interpretive framework, or increase their capacity to bear associated feelings. This achievement is observed in the healthiest level of Kernberg’s personality organization, the neurotic client, as their consistent sense of self and others allows for accurate perception of the world and subsequent adaptive solutions (Kernberg, 1968). An individual recovering from an early traumatic relationship may become aware that their current relationships have been repeating themes, and this awareness allows them to navigate future relationships free of rigidity. They are given a third dimension of awareness that enables them to make a decision every time a similar potential experience arises. In this lower end, there may be times when they vacillate between this third and second dimension, as they relapse back into patterns and are unable to sustain the third dimensional experience.

In its upper end, one may tolerate ambivalence and justify the accompanying affect by living in accordance with their ideals. As time passes, these decisions become second nature and accompanying negative affect is mitigated. We may also associate this with Maslow (1943, 1967) term self-actualization, or Jung’s (1967) concept of individuation. We might consider this position as someone who possesses high levels of insight, ego strength and self-regulation. An individual who is well aware of their value systems has the capacity to live in alignment with them. This individual can build upon their world of truth and follow a balance of their intuition and logic in moments of challenge, to such a degree that they have reached “…the most successful adaptation to universal conditions of existence coupled with the greatest possible freedom for self-determination” (Jung, 1954, p. 171). The capacity to fully operate from this third dimension implies a high level of resistance, or of the third degree, as they are capable of incorporating information and affect from previous dimensions, tolerate ambivalence, and behaviorally implement their insight and experience in accordance with who they aspire to be. The individual is active, and the majority of their decisions and actions are based on the insight and experiences gained from a successful therapeutic relationship alongside a process of continual reflection and behaviors stemming from their conclusions.

## Temporal Dynamics in Psychology

This model incorporates both implicit and explicit processes into William James’ stream of consciousness, and his characterization of the stream as emerging from “buds or drops” of experience (James, 1911, p. 1). Following Panksepp (2012), consciousness is considered in this model to be endogenous to the brain, beginning at the brain stem (anoetic consciousness). In earlier movements, flights relate to spontaneous activity in the brain, and in later movements, transitions between substantive perchings which may be characterized by an ascending recruitment of cortical regions. Perchings are culminations into substantive processes of consciousness, which are more stable in nature (Pred, 2005). We identify these flights and perchings through the lens of temporospatial dynamics between the brain and environment.

Time in this approach refers to the passage of time and consists of seven iterative movements. Importantly, movements are not unidirectional. Rather emotions and thoughts may evolve or devolve. Moreover, each movement has a transitional or transitive process before reaching one which should be considered substantive. The task of the psychotherapist is to help facilitate the transitive processes in between each substantive process. These substantive “perchings” can be remembered through the mnemonic NAP ALARM and include:

1) Neuroecological
2) Affect-Soma
3) Protothought-Image
4) Associative-Non-linear
5) Linear-Concrete
6) Abstract-Relational
7) Merging-Integration

Patients may present with psychological challenges which may be localized at any phase within the movements. These movements may be sequential but they are not necessarily hierarchical, meaning an abstract movement, for example, may not fully incorporate previous movements, though previous movements allow for abstraction to occur. This is due to the recursive nature of consciousness. An individual may know what the ideal behavior expressed may be in any particular context, but they still might not go through with it. Feedforward and feedback loops in the brain enable information to flow in many directions, such as subcortical-cortical (i.e., bottom-up), left hemisphere-right hemisphere and their corresponding inversions (i.e., top-down, right left). Similar to spatial dimensions, therapeutic action entails the incorporation of higher temporal movements. Following this model, each preceding phase is nested in every proceeding phase, temporally. Each movement labeled describes the substantive perchings, whereas each transitive portion alludes to the task of the therapist. Table 2 refers to a summary of neuroanatomical regions that have been associated to each movement, further explicated in each section description.

**TABLE 2:** Seven temporal movements and neuroanatomical correlates.

Movements	Neuroanatomy
Neuroecological	Brain stem, hippocampus, periaqueductal gray, bilateral insula, prefrontal cortex, visual cortex, cortical midline structures
Affect-Soma	Brain stem, limbic, hippocampus, amygdala, ventromedial prefrontal cortex, mesolimbic dopamine system, basal ganglia, thalamic sensory relay nuclei, parahippocampal gyrus
Protothought-Image	Precuneus, Parietal, occipital, visual cortex, posterior parietal cortex, posterior occipito-temporal cortices, anterior cingulate
Associative - Non-linear	Precuneus, insula, left middle frontal gyrus, left superior parietal gyrus, supplementary motor area, inferior frontal gyrus, temporal lobe, posterior temporal cortex, temporo-parietal occipital junction
Linear-Concrete	Amygdala, prefrontal cortex, ventrolateral prefrontal cortex, occipital gyrus, angular gyrus, culmen
Abstract-Relational	hippocampus, ventromedial prefrontal cortex, rostrolateral prefrontal cortex, the right frontotemporal region, medial prefrontal cortex, anterior cingulate cortex, posterior cingulate/retrosplenial cortex, precuneus, temporal poles, and temporo-parietal junction Hippocampus-medial prefrontal cortex
Merging-Integration	Association cortex, motor and sensory cortex, cerebellum, dorsolateral prefrontal cortex, basal ganglia

### Movement 1: Neuroecological

The neuroecological movement constitutes the initial interactions between objects/events in the world and the brain’s intrinsic activity. There are two levels within the neuroecological movement, that of the brain’s natural predisposition as a result of genetic interactions with the environment and interactions that have been formative within an individual’s development. The brain is an experience dependent organ, requiring interactions in order to form properly. This first movement is implicit in nature.

Perceptual and cognitive systems have been shaped over many generations through extensive interaction between environment and brain, e.g. natural selection, epigenetics, experiences. Such systems evolve because enhanced performance in tasks such as identifying materials, navigating environments, and detecting predators/prey led to enhanced survival and reproduction. Under a Bayesian framework, natural scene statistics and other properties of an organism’s environment (e.g., availability of prey), interact with its genes and subsequently determine the evolution of perceptual and cognitive systems. Bayesian statistical decision theory utilizes “ideal observers,” or models of optimal decision-making performance, as both a benchmark to compare to human performance as well as to be able to consider constraints within a perceptual or cognitive task. In simulations of co-evolution of a predator and prey species, Geisler and Diehl (2003) found that organisms’ perceptual and cognitive systems appear to prioritize reproductive payoff above accuracy. They have been shaped by natural selection and consequently, shape our experience.

One formulation of the Bayesian brain developed by Karl Friston is the free energy principle. According to Friston (2010), individuals as self-organizing systems seek to limit prediction errors (i.e., discrepancies between top-down predictions based on prior information and actual sensory information) by continually updating beliefs and probabilities using newly sampled data, a process known as active Bayesian inference. Models of Bayesian inference have exhibited temporal nestedness in which state transitions proceed at different rates at different levels of the hierarchy, generating outcomes over different, nested timescales (Friston et al., 2017).

In Solms’ (2020) neuropsychoanalytic model, memories serve as predictions for future event sequences and are represented by “outgoing” neurons, neurons in a permanently altered state which are only partially affected by stimuli. According to Solms, memory lies within a predictive hierarchy in which deeper layers of outgoing neurons are increasingly resistant to excitation or change from incoming sensory data. Non-declarative subcortical memory traces are more certain and resistant to change compared to declarative cortical memories because they prioritize simplicity and in turn, generalizability, over accuracy. While subcortical memory appears to reflect core homeostatic predictions and behave like reflexes, the cerebral cortex accounts for uncertainty in unpredictable situations through contextual memory; by integrating context-specific aspects of an event, the brain creates a more accurate though less generalizable model which may progressively consolidate toward the core layer of certainty and resistance.

With the primary goal of minimizing prediction errors, individuals and organisms in general may alternate between changing sensory input to match predictions (performing actions) or changing predictions to better explain incoming sensory data. Such learning already begins in infancy; through their caregiver’s predictable input, an infant can integrate sensory information into stable models of emotional and interpersonal consequences (e.g., caregiver physically soothes when infant performs action of crying). Actions as well as the generation and updating of beliefs allow them to exchange material and information with the environment while maintaining the Markov blanket boundary. For example, a hungry infant increasingly develops a differentiated sense of self as they are introduced to predictable combinations of intero-exteroceptive sensory signals and actions which facilitate breastfeeding. However, free energy (i.e., entropy) in the form of unresolved uncertainty or prediction errors may disrupt the infant’s internal secure base from which to explore their environment (Holmes and Nolte, 2019). The boundary is destabilized and they are unable to properly evaluate their environment as a separate agent in order to manipulate it to their advantage. Consequently, early traumatic events may limit future sampling of environmental information (i.e., exploration/engagement of environment) and thus hamper one’s ability to correct prior erroneous beliefs in light of new inputs. Future attachment-related psychopathology such as learned helplessness and deficient collaborative mentalizing may manifest as a result of selective informational intake and simplistic, dysfunctional internal models.

The neuroecological layer may be indirectly assessed through observable responses and neuroanatomical correlates of encoding information. Harnett et al. (2017) demonstrated positive association over time between glutamate/glutamine concentrations and post-traumatic stress severity in recently traumatized individuals. As glutamate is crucial to memory formation, alterations in glutamate networks may reflect cognitive symptoms of trauma. Higher availability of metabotropic glutamate receptors, specifically the subtype mGluR5, which mediate neuromodulatory effects of glutamate is reported in individuals with PTSD and is associated with an increase in avoidance symptoms (Holmes et al., 2017). It is possible that the higher glutamate receptor availability reflects synaptic adaptation in face of glucocorticoid signaling deficits within PTSD.

Entropy also appears to be a promising clinical diagnostic marker. Schizophrenia patients exhibited decreased temporal brain entropy in the right middle prefrontal cortex, bilateral thalamus, right hippocampus and bilateral caudate as well as increased temporal brain entropy in the left lingual gyrus, left precuneus, right fusiform face area and right superior occipital gyrus (Xue et al., 2019). A resting-state functional MRI analysis revealed that patients with Alzheimer’s disease demonstrated increased entropy in the middle temporal gyrus and precentral gyrus as well as a negative correlation between entropy and network connectivity of intrinsic brain activity (Xue and Guo, 2018). As previously mentioned in Duncan et al. (2015), perception of events as traumatic was related to higher entropy and lower levels of glutamate in the PACC. A key insight from their data was that life events were measured through whether participants subjectively perceived them as traumatic or not. For example, one subject may consider an instance of bullying as traumatic whereas another would not perceive their own experience of bullying as traumatic at all. Therefore, entropy and glutamate levels do not reflect the nature of the life event “as real” but rather spontaneous activity’s modification of one’s perception of the event.

Trauma-related symptoms can be modeled within memory dysfunction as a result of altered resting-state functional connectivity. Cracco et al. (2020) found preliminary evidence of dysfunctional spontaneous activity in adult women with experience of childhood abuse as participants showed hypoactivation of the right temporo-parietal junction during a spontaneous cognitive mentalizing task, a region commonly implicated in the individual’s “theory of mind” network (i.e., attributing states, beliefs, or perceptions to others). Using resting-state mean amplitude of spontaneous low-frequency fluctuation, Li et al. (2020) found that visual working memory was negatively correlated with severity of PTSD symptoms of earthquake survivors. For individuals with PTSD, visual information processing may be impaired and declarative memory of visual information subsequently. Symptoms such as flashbacks (i.e., visual re-experiencing of traumatic events), may occur due to altered functional connectivity related to vision. In particular, intrinsic functional connectivity between the visual cortex and cerebellum was shown to predict individual PTSD symptom severity and previous research has associated hyperactive function of visual cortex with PTSD symptom severity (Suo et al., 2020).

Spontaneous activity appears to play a critical role in continually shaping the processes of encoding and retrieving information which construct one’s sense of self and their beliefs and actions. Northoff’s neuroecological model of the brain describes spontaneous activity as the bottom most layer, formed through encoding of early life events utilizing spontaneous activity’s internal modifying power which is reflected in the second layer of inter-individual variation in entropy and glutamate. Finally, these inter-individual differences relate to the surface layer of symptomology and clinical distress observed in clients. The incorporation of temporospatial neuroscience into clinical interventions should address the client’s early hierarchical construction of bottom-up and top-down processes which influence how they relate to their environment and utilize relevant neurological markers in diagnosing a possible disorder. In contrast to models of the brain that involve passive modification, the neuroecological model recognizes the bottom layer, the brain’s ability to actively modify its own temporospatial structure through attenuation or amplification of perception of events into temporospatial memory.

## Transitive 1-2

The neuroecological layer is an underlying process behind all other functions which can be defined neurobiologically by signature patterns and rhythms of spontaneous activity. Emotional feelings and the content surrounding them operate on an implicit level. The aim of psychodynamic psychotherapy is helping make the unconscious conscious. The analysis of dreams may be particularly applicable for this phase, alongside interpretations of transferential dynamics. Insight and experience, in combination with a reduction of emotional valence of a traumatic experience, may allow previously compartmentalized information to be integrated into a coherent temporospatial structure.

More recently, dissociative symptoms which have been difficult to clinically treat have been hypothesized to be a disorder of integration, interrupting dynamic temporospatial activity at regional (e.g., insula, PACC, PCC, mPFC), network (e.g., DMN, Salience Network, Central Executive Network) and global levels (spontaneous activity; Scalabrini et al., 2020a). Subjectively, developmental trauma may result in the disconnection of self-experiences which may dysregulate the capacity of an individual to organize self-states into a cohesive overarching self (Schimmenti and Caretti, 2016). Physiological hyperarousal, spurred by consolidation of the traumatic event “threatens to overwhelm the mind’s ability to think, reflect, and process experience cognitively. This is especially true of affective dysregulation that carries the person to the edge of depersonalization and sometimes self-annihilation. Continuity of selfhood is here most truly at risk, and it is here that shame contributes its own terrible coloring.” (Bromberg, 2011, p. 23). Consequently, this can prevent the requisite transition from occurring.

Therapists and clinicians may be able to help traumatized individuals by addressing the relational verbal and non-verbal skills of the patient in the context of a secure therapeutic alliance. The therapist aims to facilitate a continuous and temporally extended and integrated sense of self and sense of relatedness, as well as an upregulation of the capacity for affect regulation. Neurologically, this process may be reflected by a reorganization of cortical/subcortical activity such that higher cortical areas (orbitofrontal cortex) can regulate, reprocess, and dampen affective arousal arising from subcortical areas (amygdala). Traditional approaches such as CBT (Kennedy et al., 2013), and psychodynamic therapy (Spermon et al., 2010) may address distressing symptoms associated with dissociation, yet these treatments alone necessitate advancement, especially for more severe cases. Adjunctive novel psychotherapeutic approaches, such as transcranial magnetic stimulation (TMS), have been beneficial particularly for treatment-resistant depression and potentially dissociation. TMS can be used to facilitate neural temporal-integration by stimulating areas such as the DMN in order to enlarge spatial and temporal scales and thus help with states of mental dissociation (Northoff and Scalabrini, 2021). Psychotherapeutic approaches designed to impact spontaneous activity have yet to be fully formed, though its inception has begun and is currently labeled spatiotemporal psychotherapy (ibid).

### Movement 2: Affect-Soma

In this movement, the individual’s cognitive functions may be recruited by one of their primary process affective experiences, or by somatic impulses. They identify with the emotion as they are not reflecting upon them. As such, they feel the emotion but there is no meta-awareness. Acting out may be a common phenomenon during this movement, for example an individual who identifies with anger may react by being physically aggressive. Another example may be a patient who has struggled with authority figures throughout his or her life and is now displaying some form of aggression toward the therapist. There is no subject-object distinction in this phase, which is why it would also fall within the spatial realm of anoetic consciousness and undifferentiated dimension.

In Panksepp’s (1998) model of emotions and affectual systems (see Table 3), he concentrates on developing ideas behind individual systems interrelated within the neuroanatomical modulates within the brain impacted by a particular affective response. This correlation examines our predecessors, our “reptilian brain,” and our developed forebrain establishing all executive functioning and everything in between. Panksepp discusses two types of consciousness: affective and cognitive. As psychotherapy evolves, a core concept in the evolution of psychotherapy is on fostering our cognitive control over our affective (or emotional) processes (Panksepp and Biven, 2012).

**TABLE 3:** Seven core emotional systems.

Panksepp’s core emotional systems	Structure	Functions
SEEKING system: Expectancy and Motivation	Ventral tegmental area (VTA), medial forebrain and lateral hypothalamus (MFB-LH), nucleus accumbens, medial prefrontal cortex via mesolimbic and mesocortical dopamine pathways Dopamine Norepinephrine Serotonin	Sensory perception Appetitive learning Addictions Emotional Needs Memory integration General arousal
RAGE system: Anger	Medial areas of the amygdala, hypothalamus, periaqueductal gray (PAG) ventromedial hypothalamic (VMH), acuate nucleus, lateral septum, neocortex(frontal executive regions), cerebellum and cerebellar cortex Gamma-aminobutric acid (GABA) Testosterone	Hormonal impacts Inhibition/Disinhibition of arousal Emotional “control” Social aggression (non-violent) Increase/decrease in aggressive response Energy depletion Sensitivity to touch Behavioral tendency toward aggression
FEAR system: Anxiety	Central amygdala, interior and medial hypothalamus, PAG in the midbrain, nucleus reticularis pontis caudalis	Autonomic nervous system Physiological and behavioral responses Arousal from pain Conditioned avoidance Anxiety (specific to fear response) Opiate receptor response Serotonin depletion or augmentation Distressing thoughts
LUST system: Sexuality	Medial region of anterior hypothalamus, interstitial nuclei of anterior hypothalamus (INAH), POA, VMH	Sexual urges and abilities Arousal Hormonal impacts Sexual bonding and competitiveness(vasopressin in males) Sexual and social motivation
CARE system: Nurturance	Anterior hypothalamus, paraventricular nucleus (PVN), dorsal preoptic area (dPOA), strial terminals (BNST), VMH	Dysfunction resulting in reduction of maternal behavior Nurturing offspring Hormonal changes (estrogen, progesterone, oxytocin) Regulation of separation distress
PANIC/GRIEF system: Separation	Paraventricular nucleus (PVN), hypothalamus, hippocampus, anterior pituitary gland	Stress hormone release (cortisol, adrenocorticotropic hormone) Energy production Separation distress control Creation of episodic memories Development of social memories and bonds Facilitates social cognition and benefits Self-regulation
PLAY system: Joy	Neocortex, thalamus, parafascicular complex, posterior dorsomedial thalamic nuclei, cerebellum, VMH, basal ganglia, amygdala, temporal lobes	Regulation of movement Arousal of emotional states Aggressive pathology Social capabilities and responses Symbolic interpretations of PLAY

Many scientists define affectual experiences as a derivative of cognitive reflection based on bodily response as opposed to responses to only brain mechanisms. Panksepp, aiming to provide foundational relevance to our affective systems, focused on ancient aspects of our minds as essential for other higher mental states, noting “to understand the whole mind, we must respond to ancestral forms” (Panksepp and Biven, 2012, p. 14) which were first to be developed in our brain’s evolutional maturity. Through a series of electrostimulation based studies, Panksepp investigated how instinctual behavior relates to emotional responses while engaging with various levels of conscious thought within affective awareness. Higher brain functioning would unequivocally fail as they are built upon a primary/affective foundation. Affects are primary experiences – emotional, instinctual affective-responsive behaviors – with visceral responses. All which are composed within several different subcortical systems within the brain (Panksepp and Biven, 2012).

Previous research presents cognition and emotion as separate entities and now we understand their integration in more depth. It is widely accepted that appraisals do not require conscious recognition. Hence emotions can be present without explicit stimuli. The mere presence of an emotion, without an obvious stimulus, involves historically based implicit memories (LeDoux, 1996). Anoetic consciousness is an affective implicit state of sensory-perceptual mental experiences. Outlined by its unreflective nature, anoetic conscious states involve an individual’s “self-experience” (Vandekerckhove et al., 2014, p. 1). This state is foundational for acquiring knowledge and memory as well as the development of consciousness (“knowing-consciousness”) which allows our memories to travel through space and time. The brain-body state within this movement of anoetic streams of consciousness depend on subcortical neural networks, thalamic sensory relay nuclei, basal ganglia, mesencephalic and diencephalic attentional and affective systems – all allowing further development of our anoetic state into a noetic state of world perception (IBID, 2014).

Anoetic affective awareness or consciousness transpire through ancestral aspects of our brain functioning. Positive emotions may reflect certain survival instincts and negative emotions may anticipate potential threats (Vandekerckhove et al., 2014). For example, Panksepp identifies neuro-coordinates within the SEEKING system associated with anoetic states of consciousness within individuals who are suffering from addictive behaviors. This driven state is involved in all appetitive behaviors motivated in stimulation of mental activity (Alcaro et al., 2021). Prior to the expansion into a noetic state of awareness, the anoetic state represents a disconnect between the self-concept in connection with the world around them or external experiences. Without the development of self-concept in a noetic state, the individual is unable to differentiate specific features of their surroundings or connect with conceptual past and present awareness. The eldest entity of our evolved brain is the medial temporal lobe inclusive of the hippocampus and parahippocampal gyrus. This area processes spatial routing and formation of abstract thinking which helps spatio-temporal and declarative memory, learning, and certain aspects of collective perception. Dysfunction within the SEEKING system is reasonably linked to addictive behavior when there is a disruption in the system’s exploration to “maintain spontaneous and unfocused attention” (Alcaro et al., 2021, p. 6). There is a disconnect between weakened intrinsic exploration and impulsive functioning within the mind-brain. Panksepp solidifies research in support of this hypothesis, indicating patients with addictive disorders exhibit alterations during their resting-state brain activity with “intrinsic functional connectivity of medial cortical structures.” (Alcaro et al., 2021, p. 6).

Neurobiological processes involved in addictive behavior revolve around the mesolimbic dopamine (MLDA) system. This system is cohesively involved in both classical conditioning and operant conditioning and is responsible for learning-associated emotional and behavioral outcomes. Panksepp and colleagues hypothesize the involvement of the MLDA system with reinforcements by limbic structures and cognitive and motor sequences (IBID, 2021). Through the learning process of the SEEKING system, its function can be narrowed down to specific boundaries within its operations – narrowing its functioning to an individual’s exploration toward a specific object or stimuli. For example, an individual who has learned to obtain a drug to achieve their desired state will continue to do so through reiteration which eventually reaches a complexity occurring without conscious thought or deliberation. An individual in recovery from drug addiction is known to still experience cravings after withdrawal symptoms disappear and act on habitual behaviors when presented with drug-associated triggers long after they restrict drug use (IBID, 2021).

Psychologists including Pat Ogden and Bessel Van Der Kolk have extensive research within client populations who experience somatic responses in their recovery post trauma. These clients are experiencing what constitutes an anoetic form of a natural bodily response within in response to a conditioned stimulus, such as a reaction to a loud noise from fireworks a war veteran would exhibit after they are reintegrated into society. There are conditioned emotional and cognitive processes that tend to disrupt an individual’s ability to function interpersonally (Ogden and Minton, 2000). For example, in a disrupted emotional state, feelings of fear may heighten the individual’s sensorimotor processes. Ogden and Minton state that in order for disruptive sensorimotor processes to be addressed clinically, we must also include the integration of the emotional and cognitive processing (IBID, 2000).

During this anoetic state and during sensorimotor processing within this movement, the client may be asked to start the integration of physical and emotional state to cultivate awareness and integrate these sensations as a way to understand their own cognitive processing. Awareness and self-regulation may not be achievable during this movement, but following this movement as the integration occurs cognitively, emotionally, and somatically. What is going on in the brain in the meantime is specific within this movement, systematically comprising various hierarchical higher and lower-level functions. The cortical (higher) levels of the brain are seen as mainly involved in our ability of abstraction, reasoning, understanding, language, and learning. Lower parts of the brain mature prior to the higher levels and are mainly associated with sensory integration and intersensory association (Ogden and Minton, 2000). The development of such higher levels is dependent on the development and functioning of the lower levels of the brain. Sensorimotor processing is foundational in the development of other areas, including integration of primitive areas (IBID, 2000).

Clients may experience an override of somatic or sensory experience. For example, from a top-down perspective, our higher cortical areas pursue our schedule for the day, optimally overriding any sensation we may feel during this period such as hunger or fatigue. “It’s as though we hover just above our somatic and sensory experience, knowing it’s there, but not allowing it to be the primary determinant of our actions” (Ogden and Minton, 2000, p. 153). The top-down and bottom-up perspectives offer two distinct directions for processing of information. The cortex offers top-down processing with cognition involved, observing and regulating the lower levels. Sensorimotor and emotionality is widely initiated from bottom-up levels of processing, fundamental to functionality (IBID, 2000).

In Damasio’s somatic marker hypothesis, the non-conscious is acknowledged through a convergence of signals which influence decision making. This concept was proposed in recognition of acute disruption in social behavior due to damage in the ventromedial sector (Bechara et al., 2000). These defects impact the ability to apply logical reasoning, maintain attention, or develop language in response to thought. This hypothesis marks crucial components in overall decision making. Since emotions are mostly occurring through somatic changes in response to transient changes within the brain, the term “somatic” is referring to internal milieu, visceral, and musculoskeletal within the somatic experience (IBID, 2000). These somatic experiences are a reply to the body’s response to regulation of experienced emotions, without direct experience in the body but in the brain’s momentary representation of the body (IBID, 2000).

These somatic markers are experienced within the ventromedial prefrontal cortex (vmPFC) and the amygdala (Damasio et al., 1996). The vmPFC links authentic knowledge and bioregulatory states, which include emotionality (Bechara et al., 2000). An integral role of emotions fall within our ability to make rational decisions quickly even in more complex scenarios. Frontal lobe damage specific to the vmPFC is known to decrease one’s ability to utilize emotions in guiding decision making processes. Given this, Damasio proposes that these changes in the body in response to certain stimuli are both a body and brain state including physiological states like increased heartbeat, changes in hormonal activity or digestive states in response to previously categorized stress. These corresponding bodily changes (somatic markers) become linked with certain stimuli over time (Damasio et al., 1996).

For example, a client who experiences symptoms of PTSD after exposure to a traumatic event may face similar situations where their brain has previously categorized fear and certain reactions are now activated in “higher-order association cortices” leading to vmPFC emotional re-activation from a previously adopted “factual-emotional set” where the brain adopts a pattern as an authentic experience when envisioning a futuristic outcome (Bechara et al., 2000, p. 297). During one phase of re-activation, called the “body loop,” the brain has adopted a specific response to a stimulus where a somatic response distinctly changes in response to the activation, then relays those changes to somatosensory cortices. In the second re-activation phase, the “as-if body loop,” the body is bypassed and the signals are sent to somatosensory structures within the brain which then adopt those patterns of re-activation. Establishment of these markers parallel to the situation qualifies the scenario deciding whether it is a positive or negative experience (Damasio et al., 1996). This hypothesis suggests that the individual’s decision making process is impacted due to the patterns associated with uncertainty of future outcomes, thus the space and time to make this decision is limited, impairing effective cognitive processing (Bechara et al., 2000).

## Transitive 2-3

Gestalt psychology often employs techniques that draw awareness to non-verbal non-conscious movements. Attention is diverted, and may be used to facilitate awareness. Psychodynamic systems may follow with a clarification, confrontation and/or interpretation of emotions at play (Kernberg, 2016). One may further use bottom-up approaches to target somato-affective processes, such as Pat Ogdens sensorimotor psychotherapy, Peter Levine’s somatic psychotherapy and Bessel Van der Kolk’s limbic psychotherapy. Other examples include (but are not limited to) dance therapy and yoga.

### Movement 3: Protothought-Image

In this movement, the individual perceives the environment through images as a result of interactions between predictive and sensory-perceptual systems. Mental imagery in one’s stream of consciousness is also now available as the parietal cortex is capable of influencing the visual cortex. Mental images may enable an individual to contain visceral emotions and responses by forming associations and giving shape to a representation which can be further explored in a clinical environment. In this sense, mental images may be considered a form of proto-thought. Inherent in this movement are somato-affective experiences alongside perception and images. Primary process emotions compose our experience but there is no discernment or discrimination of emotions. There is now some subject-object distinction. Bion (1963/2018) considered dreams to be a form of proto-thought, necessary for the functioning of the whole.

Mental imagery constitutes visual representations and accompanying sensory information in absence of an external stimulus (Blaisdell, 2019). There is evidence that an individual’s early visual cortex (V1 and V2) contributes to the ability to perform visual imagery and distinguish between different imagined stimulus exemplars (i.e., imagined variations of a reinforced stimulus) (Ragni et al., 2020). Vividness of visual imagery, however, varies widely from pervasive disorder-related hallucinations to a complete absence of mental images (i.e., aphantasia), related to hypoactivation of posterior occipito-temporal cortices and hyperactivation of the anterior cingulate (Zeman et al., 2010). Visual imagery vividness appears to be related to posterior brain regions (e.g., higher order visual cortices), but inversely related to activity in frontal regions, illustrating a possible compensatory function of frontal region activity when experiencing difficulty in generating mental images.

The ability to generate images in the individual’s “mind’s eye” may be rooted in animals’ common ability to engage in associative and causal learning. In forming predictions and causal knowledge, humans and other animals retrieve representations of associated events which inform and guide behavior. Fast et al. (2016) demonstrated that a functioning hippocampus in rats was related to their ability to behave according to previous representations of events (i.e., act in absence of a previously conditioned visual stimulus). As the hippocampus is involved in the retrieval of representations of missing events (i.e., memories), environmental cues may trigger related sensory information or outcomes such as the presence of food. As a result, animals can form novel combinations of associated representations which enable them to anticipate and simulate future events. This primordial form of mental imagery and representation may remain in our dream-thought and dream imagination. Some form of archaic mind-wandering may have already evolved before the arrival of endothermic animals (mammals and birds) as reptiles have been shown to exhibit neurophysiological correlates of REM sleep (Shein-Idelson et al., 2016). According to Jung, dreams and related thinking such as active imagination are not only the symbolic content of our inner affect but are also a type of primary process thinking in which noetic images form complexes that shape the verbal narratives which define our identity and behavior (Alcaro and Carta, 2019). However, if an individual’s respective imagery is unable to sufficiently contain their affective states, then they may be projected onto their external perceptual world such that the individual is unable to differentiate between impressions from external reality and internal projections.

The neural mechanisms of mental imagery may be compromised in certain disorders. Individuals with chronic schizophrenia exhibited lower activation of the posterior parietal cortex compared to healthy controls when performing mental imagery tasks (i.e., mental hand-rotation; Mazhari et al., 2015). Hach et al. (2014) demonstrated within depressed patients relatively lower recruitment of brain regions related to memory specificity (e.g., the hippocampus, during autobiographical memory tasks). Mental imagery may be clinically assessed in its impact on the individual’s emotions and behavior. For example, one might experience a maladaptive kind of “mental time travel” in which they visualize images of future situations that ultimately foster actual distress or danger to them. Pile and Lau (2020) found that adolescents’ experience of negative intrusive prospective imagery or flash-forward, may be associated with generalized anxiety (GAD) and depression (i.e., the impact of flash-forward is positively related to GAD and depression). Their data also suggest that the use of suppression as an emotional regulation strategy does not alter the vividness of mental imagery but rather worsens the impact of flash-forward within GAD. Consequently, clinicians working with generalized anxiety may also involve exploring suppression.

Suicide-related flash-forward may play a unique role in bipolar affective disorder. Hales et al. (2011) found that individuals with bipolar disorder relative to unipolar depression (1) had greater trait propensity to use mental imagery in general and (2) were more than twice as likely to report that suicide-related imagery was compelling in completing suicide. Such imagery was considered as both comforting and/or distressing. Suicide-related flash-forward may be experienced as comforting if the image is consistent with the individual’s goals to complete suicide or distressing if the imagery is in conflict with their ambivalence on suicide. Further development of a functional analysis of suicide-related imagery and assessment risk may provide a helpful tool for clinicians working at an image-level with clients experiencing intrusive prospective imagery of self-harm or suicide.

In one of the most commonly diagnosed disorders, depression, clinical reports of negative intrusive imagery include scenes of past early physical or sexual assault/abuse, humiliation, failure, and overwhelming sadness (Holmes et al., 2016). For example, one might visualize the loss of a loved one or being bullied/yelled at. In addition, depressed individuals appear to experience (1) less vividness in positive self-generated imagery relative to negative imagery, and (2) retrieve less specific memories or more over general memories (OGMs) when recalling positive events. This impoverished positive mental imagery may impede depressed individuals’ ability to experience positive affect and navigate interpersonal problems.

## Transitive 3-4

Existing at the border of non-conscious and conscious experience, images precede linguistic thought, and may be used to facilitate greater awareness. In psychodynamic literature images may arise as an attempt to process the potentially overwhelming impact of affect. As a consequence of this rigidity, psychological events are frozen in service of mastery. Free association in relation to representations may enable the expression of affect and somatic based signals considered to be subconscious. Examining intrusive images or the spontaneous generation of an image while experiencing a somato-affective experience (such as Cycling; Chan, 2021) may be quite productive in the pursuit of insightful experiences.

### Movement 4: Associative-Non-linear

This movement is primarily concerned with passive mind wandering and actively, an associative action that may facilitate the development of linear thought. It is creative by nature, non-linear. Free association capitalizes on this capacity and may be passively recruited by primary process affect. Associative introspection induces the exploration of content related to the Default Mode Network (DMN), a neural network dedicated to resting state activity correlated to functions such as self-referential processing, social cognition, prospection, and autobiographical recall.

Although resting-state activity may enable an individual to generate spontaneous and imaginative thoughts, it appears that aberrant functional connectivity within regions related to the DMN are related to the dysfunctional thoughts observed in some disorders. Philippi et al. (2018) demonstrated in women with varying depression histories that negative self-focused thoughts (SFT), repetitive thoughts about negative aspects of oneself (e.g., feelings of worthlessness and self-blame), were associated with increased resting-state functional connectivity (rsFC) between the DMN and the frontoparietal network (FPN). Because the DMN is related to internally focused contents while the FPN involves goal-directed behavior and externally related information, the increased rsFC between these two networks during resting-state may reflect an over-recruitment of FPN regions in order to compensate for high levels of negative-SFT that occur in depression. Similarly, major depressive disorder has been related to increased global connectivity within the DMN, i.e., abnormal strong connections with non-DMN networks (Scalabrini et al., 2020b). This increased global representation of DMN activity may represent the “enslavement” of input-output processing in which brain activity increasingly revolves around the DMN. Consequently, a depressed individual may have difficulty switching attention from self-generated thoughts toward external stimuli and from rumination to thoughtful reflection. In regards to adolescents with major depression, participants who had attempted suicide compared to those without a history of suicide attempts and healthy controls showed decreased rsFC of the left middle frontal gyrus with the left superior parietal gyrus (Schreiner et al., 2018). They also exhibited decreased rsFC between the left superior frontal gyrus and the right anterior cingulate cortex. It appears that an abnormal left fronto-parietal circuit which is involved in decision-making and choice is an underlying neural mechanism related to suicidal behavior associated with depression. Taken together, if an individual experiences a reduced ability to exert cognitive control, then such aberrant functional connectivity may allow for dysfunctional, sometimes dangerous, thoughts to pervade their experience.

How may functional affective content which motivates our regular behaviors sometimes develop into depression or ultimately suicide-related thoughts? This progression may manifest when self-generated thoughts (SGT; e.g., mind-wandering, memory retrieval), lose spontaneity and become increasingly cognitively inflexible. Patients with major depressive disorder MDD tend to engage in maladaptive SGTs such as excessive rumination and worry (Hoffmann et al., 2016). Depressive rumination involves repetitive and passive focus on causes and implications of distressing symptoms, while worry constitutes future-oriented thoughts and images in an attempt to anticipate threats. Rumination appears to be related to (1) vulnerability for developing MDD (2) duration of MDD episodes and (3) probability of relapse (Nolen-Hoeksema et al., 2008). Under a cognitive framework, early traumatic events may cause an individual to internalize negative self-referential schemas which alter their encoding of information and later retrieval of memories. Depressive rumination and worry may occur as a result of ineffectual processing and reflection. Compared to controls, depressed subjects evidenced higher levels of negative SGTs, and even positive SGT’s were rated lower and more negatively (Hoffmann et al., 2016). These results are in concordance with the notion that individuals with MDD experience a lack of positive affective content from which to explore solutions to their distressful symptoms. Sharifi et al. (2017) found increased levels of depression and anxiety among elderly individuals who exhibit deterministic thinking which were mediated by loneliness. It appears that losses or distress in personal and social functioning may foster increasingly inflexible narratives which constrain our subsequent attitudes and behaviors in the future.

Similar to depression, aberrant functional connectivity within the DMN appears to relate to dysfunctional cognitive processes in schizophrenia (e.g., association, retrieval, attribution), which may contribute to disorganization and disturbance of thought. Stegmayer et al. (2017) found that negative formal thought disorder (FTD) (i.e., poverty of thought), was associated with increased resting-state cerebral blood flow (rCBF) in regions of semantic processing in the temporal lobe. Their data suggest that patients with negative FTD may be ineffectively attempting to access words or meaning within their lexical-semantic memory. In addition, increased blood flow in the precuneus, involved in working and semantic memory, may be part of the individual’s neural effort to retrieve verbal information. Patients with positive FTD (e.g., distractibility or illogicality of thought), showed increased rCBF in the supplementary motor area, inferior frontal gyrus, and frontal lobe, suggesting that they experience increased speech production as well as difficulty in suppressing inappropriate mental activity. In a morphometric study of FTD in schizophrenia, Palaniyappan et al. (2015) found that a reduction of gray matter volume involving the striatum, insula, precuneus, and lateral prefrontal regions predicted severity of negative FTD. Interestingly, severity of negative FTD was also predicted by an increased gray matter volume in the prefrontal regions and dorsal anterior cingulate cortex. Social cognitive impairments and symptoms of schizophrenia are reported to correlate with abnormal over-activation of the posterior temporal cortex and temporo-parietal occipital junction, related to self-processing and face recognition (Yildirim et al., 2017). In addition, patients with schizophrenia exhibited increased misrecognition of facial emotions that correlated with disorganized thought, suggesting that the inaccurate or disorganized representation of another person’s mental or emotional state disrupts the integration of external contextual information and internal emotional representations. As a result, the individual with schizophrenia may experience disorganized thinking (e.g., bizarre speech, derailment, withdrawal). Because stable, accurate identification of emotional states of the self and others are important in organized thought-processing related to relationships and daily functioning, programs for individuals with schizophrenia may benefit from helping to build social cognitive skills (Horan et al., 2017).

## Transitive 4-5

Free association or the continual exploration of sensation, images, feelings and thoughts (SIFT; Siegel, 2012) eventually coalesce into linear thought as it applies to concrete thinking, which transitions into abstract coherence. Rogerian paraphrasing and reflection may assist the patient in developing more organized forms of thinking while developing therapeutic rapport. Mindfulness based approaches such as open monitoring may increase one’s awareness toward the content and process of an individual’s mind.

### Movement 5: Linear-Concrete

Concrete linear thinking describes a mode of thinking that relies heavily on what we observe in the physical world around us. The prefrontal cortex (PFC) lies as the seat of thinking, but different neural regions within the PFC are activated depending on the level of concreteness/abstraction of thought. Using fMRI, Christoff et al. (2009) investigated the neural correlates of different modalities of thinking (i.e., level of concreteness/abstraction), the results of which revealed that concrete concepts recruit more posterior PFC regions (vlPFC). These results are consistent with the developmental trajectory of the PFC; posterior regions develop first, followed by more anterior regions, mirroring the growth and progression of concrete to abstract thinking that emerges as children develop. Evidence from lesion studies also support these findings by showing that posterior PFC lesions can result in an impaired ability to perform concrete tasks (Badre et al., 2009).

Evidence pointing to more posterior PFC regions has also emerged via studies on the temporospatial dynamics of different neural regions recruited during object-recognition tasks. Wutz et al. (2018) found that concrete, stimulus-based object recognition was associated with increases in gamma-band oscillations in the vlPFC. Gamma-band oscillations have been shown to be associated with bottom-up cortical processing (Engel and Fries, 2010). Thus, concrete object recognition may be governed by bottom-up processing, involving the vlPFC which receives direct input from the inferior cortex via the ventral stream (i.e., the “what” stream). Further, a meta-analysis of neuroimaging studies on the neural representations of concrete concepts found that concrete concepts more heavily recruited the perceptual system (Wang et al., 2010). More specifically, thinking in concrete concepts consistently activated the left superior occipital gyrus, the angular gyrus, and the culmen. These findings suggest concrete conceptual representation and concrete linear thinking may involve mental imagery vis-a-vis the perceptual system.

Developmental psychologist Jean Piaget, known for his work on child development and cognitive development, delineated cognitive developmental stages through which children mature through as their cognitive functions develop and grow in capacity over the life span. The ability to engage in concrete linear thinking roughly corresponds to what Piaget termed as the “Concrete Operational Stage” (ages 7–11 years old). As children reach this stage, they develop the capacity for logic and reasoning, and they gain the capacity to organize objects into hierarchies of classes and subclasses. However, logical thinking and mental organization is still limited to concrete information they can perceive directly (Berk, 2018). This can also be conceptualized as stimulus-bound thinking, in that thinking is directly related to the stimulus by which the thought is provoked.

Linear concrete thinking also includes what Jaak Panksepp termed as *tertiary-processes*, which can be thought of as higher-order cognitions/thoughts that are elaborated by medial-frontal regions and permitted by the massive encephalization as humans evolved from primates (Panksepp, 2010). Such higher-order, neocortically based cognitive processes rest on a foundation of *primary-process emotions* (basic-primordial affects, sub-neocortical) and *secondary-process* emotions (learning via basal ganglia, limbic-based). The blending of *primary* and *secondary* process emotions and affects with a linguistically based neocortical analysis allows for an elaborated interpretation of experience, a conception of a felt-experience that can be put into words and given meaning (Davis and Montag, 2019). For example, an individual in a state of hunger will start to formulate emotional-thoughts related to their hunger (e.g., “I feel hungry”), and may begin to virtually simulate ways in which food can be attained. Such processes are cortically elaborated cognitions pertaining to felt affects. The ability to linguistically describe felt affects can also serve a positive function, correlating with a decreased amygdala response and an increase in right prefrontal activation (Cozolino, 2014).

In a clinical setting, maladaptive concrete linear thinking and feeling may manifest when the patient’s thoughts are solely populated by current mood congruent ideas and feelings. This often occurs in the context of Borderline Personality Disorder (BPD), a mental disorder characterized by unstable moods, behaviors, and relationships. Specifically, a patient with BPD may engage in what is known as dichotomous thinking, a form of concrete linear thinking in which an individual thinks in terms of binary, all-or nothing categories. Individuals with BPD have been shown to engage in higher levels of dichotomous thinking when making appraisals and interpretations of others compared to non-BPD patients (Arntz and ten Haaf, 2012). For example, an individual with BPD may oscillate between feelings of love and feelings of intense hatred toward a significant other, depending on their current mood and situation. Such dichotomous thinking and cognitive appraisal of the significant other lacks the nuance and complexity characteristic of healthy social cognition, which would be more balanced and well-rounded. In addition, there is a marked absence of abstract, temporally based thinking which would enable the patient to think of all aspects of the relationship at once, rather than only positive or negative aspects as they appear in the current moment. Linear concrete thinking in this manner may keep the client trapped in the present moment, unable to think about aspects of the relationship that occurred in the past or that could potentially occur in the future. High emotion sensitivity combined with a bias toward negative emotion may result in diminished cortical activity, causing deficits in cortically mediated emotional regulation, and an upregulation of more primitive, limbic emotional-based responses that are triggered by the present emotion-laden moment. Clinicians may find it helpful to encourage the individual to reach into their memory and take into account other past experiences in order to build a more nuanced interpretation during emotionally charged situations.

Another clinical illustration of maladaptive concrete linear thinking can be seen in individuals presenting with substance use disorder, for whom thought processes related to obtaining and using a certain substance often dominates their mental space. Such individuals may find themselves constantly thinking about the substance in question, especially in situations in which the substance in question is present. Thoughts about the substance can also be triggered by environmental cues. Depending on the degree of dependence, some clients may think about a substance to the exclusion of almost every other area of their life, demonstrating the strong mental grip that substance abuse can have on a client’s mental life. As a consequence of tolerance and withdrawal, such individuals may experience strong urges to use, resulting in *tertiary-process*, affect-congruent thoughts that revolve around the substance.

## Transitive 5-6

The transition from linear thoughts to abstract thought entails the necessity for the individual to develop multiple perspectives about singular events, beyond what is physically and temporally available. There is a connection to be made as information is assimilated or accommodated into pre-existing frameworks of knowledge. Approaches such as mentalizing affords the patient the capacity to begin incorporating other perspectives beyond their own. Logotherapy in the form of externalized thought and dereflection may enable sufficient mental space for a client to imagine novel approaches (Baumel and Constantino, 2020). Socratic questioning in cognitive behavioral therapy may also be beneficial. New questions facilitate the development of new paradigms, increasing the complexity and veridicality of a concrete perspective. Furthermore, the introduction of thought records provides a structured approach allowing the individual to have less biased perspectives. Journaling can also be used as an effective intervention to facilitate the emergence of divergent perspectives and emotional development.

### Movement 6: Abstract-Relational

As individuals mature into adolescence and adulthood they develop the ability to form and employ abstract conceptual thought processes in order to build abstract models of the world and apply those models to various situations. Abstract concept formation entails the ability to extract commonalities and distinctions across a set of experiences in order to structure and organize knowledge. Neural structures critical to concept learning include both the vmPFC and hippocampus, which have been found to form a functional alliance when encoding new information that overlaps with prior experiences (Preston and Eichenbaum, 2013). This functional circuit is thought to underpin both the acquisition of conceptual and abstract knowledge and serve as a foundation for conceptual decision making (Kumaran et al., 2009). Hippocampus functioning is thought to facilitate pattern completion, pattern separation, and memory integration (Mack et al., 2018), while the vmPFC mediates the active integration and evaluation of such associative information provided by the hippocampus. The vmPFC may also play a further role in down-weighing irrelevant stimuli features and up-weighing relevant stimuli features to efficiently categorize and structure new information into existing abstract and conceptual models (Mack et al., 2019). The rostrolateral prefrontal cortex (rlPFC), one of the final neural regions to reach maturity in humans and also one of the largest cytoarchitectonic areas in the brain (Dumontheil, 2014), constitutes one of the main neural regions supporting higher order abstract thought. It appears to support the ability to temporarily disengage from space and time, which allows for mental time-travel, social cognition, mentalization, and theory of mind.

These processes also correspond to Panksepp’s *tertiary-process* emotions as prefrontally elaborated higher order cognitions and include memory-related cognitive functions such as “Episodic Memory.” “Episodic Memory” entails the encoding of specific information of a particular occasion within a particular context, the reactivation and explicit representation of which form the foundation on which a continuous and temporospatial sense of “self” develops. This capacity permits a deep self-consciousness and meta-cognition, allowing an individual to mentally represent themselves as a continuing existence embedded in specific episodic contexts. Also permitted is the ability to escape the phenomenal present vis-a-vis mental time travel, directed to past events, to potential opportunities in the present time, and to future, “prospective” possibilities. Such autobiographical self-processes engage widespread networks within the right frontotemporal region, including the hippocampus, related medial temporal lobe structures, mPFC, ACC, posterior cingulate/retrosplenial cortex, precuneus, temporal poles, and the temporo-parietal junction (Vandekerckhove and Panksepp, 2011).

Closely related to the process of abstract thought formation is a notion that Piaget first introduced called “schemas” (Piaget, 1926). Schemas can be conceptualized as superordinate knowledge structures that individuals use to organize and categorize information in order to facilitate the perception and organization of new knowledge. Such structures dynamically evolve with new experiences and memories, and individuals can either assimilate new knowledge into an extant structure or modify their schemas to accommodate new knowledge that is inconsistent with the current model. Research into schema instantiation and reformulation has implicated the vmPFC as a key brain area involved in schema networks, interacting with the hippocampus to activate prior knowledge and assess the resonance between incoming information and existing schematic representations (Gilboa and Marlatte, 2017). The new schematic representation then becomes instantiated as a schema and integrated as a new memory, which is underpinned by hippocampus-mPFC theta-band coupling (Backus et al., 2016). Evidence has also emerged pointing to the anterior cingulate cortex as a structure critical for schema expression and assimilation (Wang et al., 2012), as well as mental flexibility (Mogadam et al., 2019).

The ability to form schemas and engage in higher order abstraction corresponds to a stage of cognitive development that Piaget termed the “Formal operational stage” (11–16 years old and onward). At this point, children develop the capacity for abstract and systematic thought, higher-order logic, and metacognition. Logic is no longer limited to concrete information, and children begin to develop the capacity for scientific thinking (Berk, 2018).

In a clinical context, neurodevelopmental disorders such as Autism Spectrum Disorder (ASD) may result in an impaired ability to engage in abstract thinking. Many individuals diagnosed with ASD experience limitations in their ability to think abstractly and instead rely more heavily on concrete thinking in order to solve problems and navigate the social world. For many such individuals, thinking is limited to what is in front of them, and they are often unable to generalize information into concepts. A study by Mogadam et al. (2019), using Magnetoencephalography (MEG), found that children diagnosed with ASD showed a stronger reliance on the posterior parietal cortices to complete a mental flexibility task compared to controls, suggesting that their ability to think abstractly to solve mental flexibility problems relies more heavily on the perceptual system, which is typically recruited when individuals engage in concrete linear thinking. Empathy, creativity, and flexibility may be impaired in individuals experiencing deficits in abstract thinking, who instead rely more heavily on what they can currently perceive to solve problems, interact with others, and engage with the world. Creative cognition appears to underlie response inhibition that proactively prevents previous responses or behaviors from interfering with novel ideation. Beaty et al. (2016, 2017) demonstrated that response inhibition requires dynamic interactions of large-scale brain systems, in particular, between the default mode and executive control networks. While these two networks generally have an antagonistic relationship, they dynamically interact to enhance creative cognition.

Abstract thinking, and in particular social cognition, may also depend on the brain’s motor system. Based on empirical findings, Khalil et al. (2018) propose a multilayered neural network model of ASD that includes the motor neuron system (MNS; motor system, basal ganglia, insula), and a “mind-reading network” (prefrontal cortex, anterior cingulate cortex, temporoparietal junction). The MNS is critical for understanding and imitating the behavior of others, whereas the “mind-reading network” allows for reasoning about others’ mental states, social decision making, and cognitive perspective taking. Under this framework, observation and imitation of behavior acts as a base layer of information that is elaborated into higher-order functions such as theory of mind skills and complex social cognitions. Khalil and colleagues contend that dysfunction of the MNS and its dynamical interaction with the “mind-reading network” may underpin the motor and social cognition based symptoms of ASD, resulting in deficits in action imitation, learning motor actions, and social cognition.

Maladaptive abstract thinking may also take the form of excessive rumination, often seen in patients diagnosed with depression. These patients may constantly ruminate on negative thoughts/emotions and engage in negative self-talk and self-criticism, finding it difficult to think more concretely and in the present moment. Abstract rumination is a common thinking process reported by depressed individuals (Watkins, 2016), which typically involves repeated thinking about higher-order aspects of a situation. This can take the form of reasons for and implications of a situation, and can include over-focusing on the meanings and consequences of one’s negative emotions. For example, abstract rumination can take the form of thoughts such as “what is wrong with me” and “why can’t I handle this better.” Dey et al. (2018) examined the extent to which high dysphoric and low dysphoric individuals engaged in abstract thinking while completing a decision-making task, and found that high dysphoric participants demonstrated more abstract thinking and worse outcomes on decision-making measures before and after they made decisions about both personal and hypothetical scenarios. They also found an association between depressive symptoms and longer task completion time when these individuals engaged in abstract thinking, relative to concrete thinking. These results suggest that abstract thinking in depressed individuals could contribute to decision-making difficulties, and that facilitating the use of concrete thinking may reduce these difficulties. Watkins et al. (2011) found that when people with depression were asked to think about a recent upsetting event and encouraged to break down the event into concrete details and consider how those concrete details influenced the outcome, depressive symptoms (e.g., rumination, worry, etc.) were reduced. In this context, concrete thinking may be beneficial because it encourages individuals to vividly imagine an event happening in the present moment and prompts them to confront it. Watkins et al. (2009) developed an intervention called concreteness training (CNT), which assists patients in stepping out of abstract thinking styles and stepping into more concrete ones.

Dysregulation of self-processes may also manifest as an unstable mental self. For example, social anxiety disorder (SAD) is characterized by intense feelings of fear and anxiety related to social situations, excessive self-attention, and somatic interoceptive sensations. SAD has been linked to hypoconnectivity within the DMN and to hyperconnectivity between the DMN and the amygdala and salience network during the resting state. This pattern of neural activity is thought to reflect a predisposition to emotional dysregulation and decreases in self-reflection and social cognition during resting states, which Angeletti et al. (2021) describe as a trait feature of an “unstable social self.” During SAD-sensitive situations, this “unstable social self” is abnormally aggravated, inducing hyperactivity between the DMN and the amygdala and salience network.

## Transitive 6-7

There exists a subtle but important difference, when considering abstraction and the subsequent phase termed merging. The difference is the employment of abstractions; more specifically into behavioral manifestations of insight and awareness that have been achieved. An example of this from a psychodynamic perspective might be an individual suffering from maladaptive relational patterns. After treatment, they may become aware of the pattern’s historical development and function, plan for the future and exercise self-regulation in moments of vulnerability. This encapsulates the idea of working through. From a CBT perspective, behavioral experiments, such as “acting as if”, may be implemented and then consequences examined. As evidence increases for potentially beneficial behaviors, abstractions become verified, consolidated and embodied. From an ACT approach, this would entail the identification of an individual’s value system, and the pursuit of living a life more in accordance with this system via identifying and applying novel behaviors.

### Movement 7: Merging-Integration

Movement 7 refers to a stage in which all prior layers are now integrated and abstractly related to each other, and tested out in reality. At this stage, the goal is to use newfound conceptual experiences and apply them in reality, which will facilitate their imprinting in implicit systems. By using newfound conceptual experiences and applying them to the real world, the individual will start to accrue evidence that either supports or disproves conceptual experiences.

The facilitation of conceptual knowledge into embodied real-world knowledge happens as top-down processing from higher cortical areas converges with bottom-up processing from sensory/perceptual areas, as our pre-existing conceptual models converge with incoming perceptual and experiential information to integrate, synthesize, and construct new representational models. This process of continual refinement of mental representational models via experience corresponds to a contemporary framework of neural functioning that views the brain as a predictive inference machine, always working to optimize probabilistic representations of the world by comparing incoming sensory input to its own extant representational models (Friston, 2010). Conceptual learning and the formation of ever-more predictive abstract models of reality can be conceptualized as a process of continual refinement in which incompatible sensory/perceptual experiences prompt us to modify our current schemas to make better sense of the world. Alternatively, we can alter our relationship to the environment such that incoming sensory/perceptual information is compatible and can be assimilated into our current schemas. Conceptual learning under this framework occurs as a consequence of iterative feedforward and feedback loops unfolding in the context of brain-environment relationships.

At this stage, a clinician might assist an individual to live in accordance with all of their inner self-processes (i.e., all of the previous layers). Chan (2021) defines this state as “Embodied Symbiosis”: “the relating of the conscious self-process with complex and primordial self-processes in service of experiential truth and meaning” (p. 235). Embodied Symbiosis results in the harmonization of both conscious and unconscious processes, resulting in novel conceptual paradigms and new emotional experiences that can be embodied and tested out in the real world. In a clinical setting, it is typical for clients to present with a history of cyclical maladaptive patterns in relationships. From a psychodynamic perspective, these relationships may be re-enacted in the therapeutic relationship, taking the form of rupture and repair. As insight and a corrective emotional experience is acquired, there is the expectation that the client will begin shifting their capacity to relate to others through the new internalized therapeutic relationship. To bolster this process, it may be beneficial to implement a behavioral experiment, in which the clinician can encourage and guide the client into testing out novel ways of relating to others.

Key to the corrective therapeutic process is the quality of the therapeutic relationship which has been found to be a critical ingredient of psychotherapy and the largest factor mediating positive therapeutic outcomes (Koole and Tschacher, 2016). A strong therapeutic alliance, characterized by empathy, trust, and mutual understanding, may facilitate what Scalabrini et al. (2018) term “relational alignment”, a base-layer of self-processes given by the first relational encounter with a caregiver and instantiated as a consequence of continued interactions with a caregiver and their capacity to attune with the mind-brain of the infant in the context of a secure attachment and safe environment. Attunement with the infant facilitates the process by which their mind-brain becomes relationally aligned with the world, both in time and space. Disruptions in “relational alignment”, for example through trauma or the lack of nurturing attunement, may lead to dysregulation of self-other processes and the development of personality pathologies. A strong therapeutic alliance promotes relational healing by fostering the creation of a healthy relational alignment between the therapist and patient in a safe and secure environment, allowing for the development and maturation of healthier self-processes.

One of the ways relational alignment can be (re)-equilibrated in the context of the therapist-patient dyadic relationship is through what Stern et al. (1998) define as “now moments.” “Now moments” refer to an emergent property of the complex, dynamic therapeutic process, representing moments that challenge the habitual therapeutic process (i.e., the way the particular therapeutic process had been operating so far) and open the space for a potential transition to a new intersubjective and relational context. These moments represent opportunities for non-linear jumps in the therapeutic process, creating anxiety for both the therapist and patient as they are both on unfamiliar ground. During this unstable moment, the therapist and patient can either resort to habitual ways of interacting, or can introduce novelty into the relationship, accompanied by alterations in the intersubjective environment. In turn, should the “now moment” be seized upon, therapy resumes with a new “way-of-being-with-the-other.”

## Temporospatial Grid

When combined, we have a series of temporal movements matched to dimensions of space. These movements operate through iterative cycles. As soon as thoughts and emotions are integrated to high levels of maturity they eventually (re)consolidate, moving from explicit to implicit modes of functioning. The undifferentiated (U-D) dimension of space is the ground on which everything else functions. It is completely relational and paired with the neuroecological layer. These dimensions are continually defined and redefined, as one builds upon their narratives that develop into different relationships with themselves and the world. This grid should not be used to define the entirety of an individual, as there are varying degrees of development within the biopsychosocial-existential spheres. It is useful with particularly salient challenges, or in classical terms the presenting problem.

## Case Example

The following is a brief example of how this grid may be used to guide practice. Sessions are generally not as “clean” as what is about to be presented; nonetheless, it is an example that can clarify direction. Many times, one may find a problem lies in a transition between movements, or dimensions, in which case, one may simply mark an X between the dividing lines. The question to ask oneself is, “Where in the developmental process of emotions and thoughts is the client operating from in relation to their presenting problem(s)?”

A 40-year-old, married Hispanic male presented with a propensity to cheat. He has three children. In addition, his medical history was unremarkable. He arrives at the behest of his wife who desires to maintain the relationship. He also notes his interest in continuing his marriage. Initially, he does not see cheating as a problem. He repeats “this is just how men are, women will never understand.” He could not provide a narrative about why he cheated other than simply claiming that when a biological urge arose, his job was to satisfy it. At this point an X can be marked on the spatial dimension UD and the neuroecological temporal movement (TM1). He was operating from attachment patterns he was unaware of, and could see no substantive consequence of his actions.

In another session, the client arrived and revealed that he met somebody new, and that he was interested in pursuing her. When asked to share any bodily or emotional experiences, he revealed high levels of lust and concluded that “when I am with her, I feel the impulse, and am unable to think and control my actions.” While talking about her, he began to feel them once more. He was asked to “sit with the experiences, and share any sensations he was having.” More minute experiences of lust were uncovered, and previous bodily experiences he was not aware of were brought to his awareness (TM2 and UD). After a few minutes of processing sensations, they passed. As the patient continued to undergo psychotherapy, he was able to trace back his belief to how his father cheated on his mother and she simply accepted this. When asked to share any other spontaneous thoughts, he recalled old memories, such as reinforcement from his friends and moments his father expressed pride in his capacity to maintain his relationship alongside the quantity of extramarital affairs. He began to consider how his past was influencing his present, and began experiencing psychological incongruence (but did not know why), though he continued to cheat due to a failure to regulate his strong impulses (TM4 and 1D).

**Table:** 

Temporospatial Grid	Undifferentiated Dimension (U-D) Non-Conscious -Non-duality -Attachment -Implicit, Body, Autonomic System -Secondary Emot. -Passive	1-D Anoetic Consciousness - Primary Emot. - Preconscious - Acting out, projection, transference -Passive-Active	2-D Noetic consciousness -ToM, self-rep -Thinking -Semantic -Insight w/o application -Active-Passive	3-D Autonoetic consciousness -Meta-cog. -Mentalizing -Self-regulation -Explicit Mem. -Active
Temporal Movement 1 (TM1) Neuroecological				
Temporal Movement 2 (TM2) Affect-Soma				
Temporal Movement 3 (TM3) Protothought-Image				
Temporal Movement 4 (TM4) Associative-Non-linear				
Temporal Movement 5 (TM5) Linear-Concrete				
Temporal Movement 6 (TM6) Abstract-Relational				
Temporal Movement 7 (TM7) Merging-Integration				

The client’s narrative began to develop, but he was still basing his actions on physical stimulation being a necessity for stability and he was unable to think beyond his immediate environment (TM5). The therapist decided to engage his theory of mind. She begins to ask questions regarding the effect that his behaviors may be having on his wife. His responses were self-centered at large, responding that she should not care and just recognize that this is who he is. When asked how he would feel in her position he divulges that his reaction would be quite negative. The process of questions led to the examination of her intentions of sending him to therapy, her reactions to her discovery of him cheating and consideration of her as a human being. He slowly begins to incorporate her experiences into his own. He begins to feel guilt when he cheats although he continues to do it. He begins to reduce the amount of cheating as a result of the guilt and thoughts of his family. He is taught mindfulness-based practices to assist with tolerating the compulsive urge to seek gratification outside his relationship. He continues to cheat but with less frequency (TM5 and 2D).

After several sessions, the client attempted to seek out another female therapist. He justified this by sharing that while in the past he was at peace with his activities he is now struggling and feeling guilt (regression to TM5, 1D). Through several sessions, transferential dynamics were explored in depth, emotions processed and interpretations implemented. He resonated with the idea that he was “cheating” on his therapist whom he saw as a mother figure, and began to see himself as a “prisoner to” his “past,” and felt motivated to “free” himself. He also began to recount the feelings he had as a child when he first found out his father was unfaithful (TM6, 2D).

After gaining a greater understanding of his relational patterns, an activity was given using ACT techniques to identify his values, which led to an incongruence between his behaviors and beliefs system. He began to recognize how his family culture differs from that of society at large, and that of his wife. In tandem, his desire to live a life oriented toward satisfying pleasures was reasoned to be an ineffective route and that cheating and the effort necessary to successfully do so frequently impacted his productivity in life. As incongruence grew, with the incorporation of his wife’s perspective, his children’s future, and his desire to respect himself, his mindfulness practices increased in frequency, duration and intensity. He progressed to the point whereby he was capable of tolerating many of his sexual urges. With insight, a greater sense of self and others, and increasing the power of descending inhibitory pathways, he gained the ability to subvert the influence of antiquated operating systems. Although he continued to deal with “temptations” he was capable of pausing himself, reflecting on his values and acting in accordance with them. At the end he reported an enhanced sense of peace and a much-improved relationship with his wife. At this point, an X can be marked on TM7 and 3D.

## Conclusion

This *Temporospatial Model* is a novel framework guided by neuropsychological studies, the purpose of which is to help clinicians become more informed about when certain techniques may be best used. The techniques mentioned in this article are not exhaustive by any means and only serve as examples that may provide assistance. Energy and information are metabolized through a variety of phases, before reaching behavior. The quality of psychoneurophysiological “metabolic” activity rests on a spectrum and will require continual modification as new context-dependent adaptations are needed. From this perspective, the therapist’s responsibility is to facilitate experiences occurring in each movement, the goal of which is to achieve increasing levels of integration and well-being. This model takes into account the idea that individuals exist in a continual relational process of becoming, serving as a tool that clinicians can use to track progress and help conceptualize and inform techniques. With time, neuroplastic changes impact the structure and function of the brain which furthermore alters electromagnetic activity being emitted. Clients become more flexible, adaptive, coherent, energetic, and stable (FACES; Siegel, 2012). The therapeutic process itself is consolidated and internalized, and clients become more creative and resilient toward challenges in the future.

## Data Availability Statement

The original contributions presented in the study are included in the article/supplementary material, further inquiries can be directed to the corresponding author/s.

## Author Contributions

AC conceived the theory and core ideas, contributed to the guidance, and drafted the abstract/intro, spatial, and temporal sections, transitive sections, conclusion, and large scale integration of writing and edits. GN contributed to the temporospatial domains, edits, integrating relevant research, and guidance. RK, JO, and KW drafted the temporal movements. All authors contributed to manuscript revision, read, and approved the submitted version.

## Conflict of Interest

The authors declare that the research was conducted in the absence of any commercial or financial relationships that could be construed as a potential conflict of interest.

## Publisher’s Note

All claims expressed in this article are solely those of the authors and do not necessarily represent those of their affiliated organizations, or those of the publisher, the editors and the reviewers. Any product that may be evaluated in this article, or claim that may be made by its manufacturer, is not guaranteed or endorsed by the publisher.
